# Sleep duration time and human papillomavirus infection risk: The U-shaped relationship revealed by NHANES data

**DOI:** 10.1371/journal.pone.0301212

**Published:** 2024-04-05

**Authors:** Huangyu Hu, Yue Wu, Min Zhao, Jiaqi Liu, Ping Xie

**Affiliations:** 1 Hospital of Chengdu University of Traditional Chinese Medicine, Chengdu, China; 2 Acupuncture School of Hospital of Chengdu University of Traditional Chinese Medicine, Chengdu, China; 3 Sichuan University West China Second University Hospital, Chengdu, China; Ordu University, TURKEY

## Abstract

**Purpose:**

This study aims to investigate the relationship between sleep factors (sleep duration time [SDT] and obstructive sleep apnea [OSA]) and human papillomavirus (HPV)/high-risk HPV(HR-HPV) infection, utilizing data from the National Health and Nutrition Examination Survey (NHANES).

**Methods:**

We conducted a cross-sectional analysis using NHANES data, focusing on SDT and OSA’s association with HPV/HR-HPV infection. The primary statistical methods included weighted multivariate linear regression and logistic regression to assess the association between SDT, OSA, and HPV/HR-HPV infection. The study employed restricted cubic splines (RCS) for evaluating potential non-linear relationships between SDT and HPV/HR-HPV infection. Subgroup analyses were conducted. Interaction terms were used to examine the heterogeneity in associations across different subgroups.

**Results:**

The study identified a U-shaped relationship between SDT and HPV infection. Specifically, 7 hours of sleep was associated with the lowest risk of HPV infection. In comparison, SDT less than 7 hours resulted in a 26.3% higher risk of HPV infection (Odds Ratio [OR] = 1.26, 95% Confidence Interval [CI]: 1.029, 1.549), and more than 9 hours of sleep showed a 57.4% increased risk (OR = 1.574, 95% CI: 1.116, 2.220). The relationship between SDT and HR-HPV infection was significant in the first two models, but not in the fully adjusted model. No significant interaction was found between sleep duration and other covariates. There was no association between OSA and HPV/HR-HPV infection.

**Conclusion:**

The study underscores the complex relationship between sleep duration and HPV infection risk, suggesting both very short and very long sleep durations may increase HPV infection likelihood. The findings highlight the need for further research to explore the biological mechanisms underpinning this association and to consider broader population groups and more precise sleep assessment methods in future studies.

## Introduction

Human papillomavirus (HPV) infection poses a significant challenge in global public health, profoundly impacting individual health and socio-economic aspects [1]. HPV is categorized into low-risk and high-risk types; the low-risk type is mainly associated with skin warts, while the high-risk type is related to serious diseases such as cervical cancer, vaginal cancer, and anal cancer [2–4]. It is estimated that in 2018, hundreds of millions of people worldwide were infected with HPV, with over 14 million new infections in the United States alone. Particularly in the United States, the genital HPV infection rate among women aged 18 to 59 is estimated to be as high as 43%, and the health burden of HPV infections in women is significantly higher than in men [5, 6]. Notably, most HPV infections can be naturally cleared by the host’s immune system within 2 years, but approximately 10% of infections develop into persistent infections [7].

Sleep is an active and essential physiological process in life, crucial for human health and well-being. In recent years, sleep disorders have been recognized as a significant issue in public health, playing a key role in the prevention and management of diseases [8]. Generally, the optimal sleep duration is 7 to 9 hours, but according to recent studies, people’s sleep duration has been decreasing over the past few decades [9, 10]. In the United States, only about 48% of adults report that their usual sleep duration falls within this ideal range [11]. It is well-known that poor sleep habits can have extensive adverse effects on the human body, such as being closely associated with diabetes, Alzheimer’s disease, cardiovascular diseases, and may also affect the development and progression of HPV infections [12].

Adequate sleep plays a crucial role in maintaining immune function, affecting both the innate and adaptive aspects of our body’s defense system [13]. Particularly, the interaction between sleep and the immune system is vital for understanding the occurrence and persistence of HPV infections [14]. However, current research on the relationship between sleep factors and HPV infection is relatively scarce. To explicitly explore the hypothesized relationship between sleep duration, obstructive sleep apnea, and other sleep factors with HPV infection, we conducted a comprehensive cross-sectional study using data from the National Health and Nutrition Examination Survey (NHANES). In this study, we detailed the sample selection, data collection methods, and statistical analysis process to ensure the reliability and validity of the results. Through this research, we aim to fill a knowledge gap in the existing literature and provide valuable insights for the field of public health.

## Methods

### Data sources and study population

**Fig 1:**
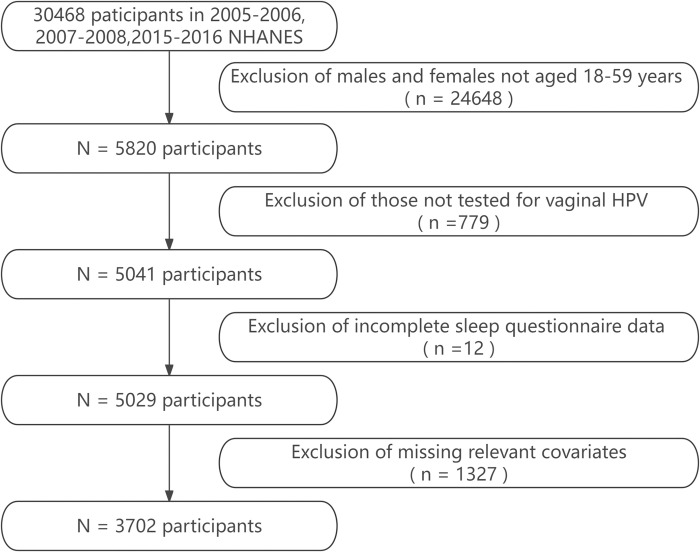
Flowchart of participant selection.

NHANES database was utilized for the collection of all data. The NHANES study has been approved by the United States ethics committee, and informed consent was obtained from each participant. This is a population-based cross-sectional survey. We selected specific data from three two-year cycles of NHANES (2005-2006, 2007-2008, and 2015-2016). These specific years were chosen because data on obstructive sleep apnea (OSA) were available only in these three cycles. The inclusion criteria for the study population were as follows: (1) women aged 18 to 59 years; (2) underwent genital HPV testing; (3) had complete sleep questionnaire data. Exclusion criteria included: (1) being pregnant or uncertain about pregnancy status; (2) missing key covariates. These criteria were selected to ensure data completeness and relevance. Ultimately, 3702 women were included in the study dataset. The data selection process is shown in Fig 1.

### Dependent variable

In this study, HPV testing data targeted participants aged 18 to 59 years in a public database. To obtain accurate HPV infection data, we used the Roche Linear Array HPV Genotyping Test. The reason for choosing this test is that it can accurately distinguish and detect multiple HPV types, totaling 37 (including 6, 11, 16, 18, 26, 31, 33, 35, 39, 40, 42, 45, 51, 52, 53, 54, 55, 56, 58, 59, 61, 62, 64, 66, 67, 68, 69, 70, 71, 72, 73, 81, 82, 83, 84, 89, and IS39). We defined a positive result for any type as an HPV infection. Specifically, if the positive types include 16, 18, 31, 33, 35, 39, 45, 51, 52, 56, 58, or 59, it is defined as a high-risk HPV (HR-HPV) infection.

### Independent variable

In this study, data on Sleep Duration Time (SDT) were collected based on specific questions from two different survey cycles. Between 2005-2008, SDT data were collected from the question ’SLD010H: How much sleep do you get in hours?’ in the Sleep Disorders Questionnaire, while in the 2015-2016 cycle, it was based on the question ’SLD012: How much sleep do you usually get at night on weekdays or workdays?’. Based on the recommendations of the American Academy of Sleep Medicine (AASM) and the Sleep Research Society (SRS), sleep duration was categorized into short sleep (<7 hours/night), moderate sleep (7-9 hours/night), and long sleep (>9 hours/night) to assess the impact of these different sleep patterns on HPV infection [8]. Regarding OSA, its definition was based on three questions, including: (1) snoring 3 or more nights per week; (2) gasping, panting, or stopping breathing 3 or more nights per week; (3) feeling drowsy 16-30 times per month even with more than 7 hours of sleep each night, on weekdays or work nights. Under these criteria, participants meeting any one of the symptoms were considered positive for OSA symptoms.

### Covariates

In this study, various covariates were used based on previous research and clinical practice. The covariates used included age, race, education level, marital status, poverty-to-income ratio (PIR), body mass index (BMI), smoking status, alcohol consumption, age at sexual debut, contraceptive use, and hormonal drug use. These covariates were obtained from the NHANES database’s demographic data, examination data, reproductive health questionnaires, sexual behavior questionnaires, smoking questionnaires, and alcohol consumption questionnaires. Based on alcohol consumption, drinking history was categorized as never (< 12 lifetime drinks), former (≥ 12 drinks in 1 year but no drinks in the last year or ≥ 12 lifetime drinks but no drinks in the last year), mild (≤ 1 daily drinks in the last 12 months), moderate (2 daily drinks in the last 12 months), or heavy (≥ 3 daily drinks in the last 12 months). Smoking status was classified as never (< 100 lifetime cigarettes), former (> 100 lifetime cigarettes but not a current smoker), or current.

### Statistical analysis

In our study, data analysis followed NHANES analysis guidelines and was conducted using weighted methods. In the NHANES database, weights include interview weight (wtint2yr) and examination weight (wtmec2yr). The choice of weight depends on the specific variable of interest in the analysis. Since mobile examination center (MEC) samples are a subset of interview samples, we chose to use the combined MEC exam weight for this analysis. The reason for using weighted analysis is that these weights are designed to extrapolate survey results to the non-institutionalized civilian U.S. population, thereby increasing the representativeness of the study results [15].

Continuous variables in baseline information are described as mean and standard error (SE), which helps to accurately present the central tendency and variability of the data. For continuous variables that follow a normal distribution, we use one-way analysis of variance (ANOVA); for non-normally distributed variables, we use the Kruskal Wallis test. For categorical variables, we analyze using the chi-square test and describe them in percentages. In this study, we used a weighted multivariate logistic regression model to analyze the relationship between SDT and OSA with HPV/HR-HPV infection. The models are divided into three: model1 without any variable adjustment to assess the original association; model2 adjusts for age and race to control the impact of these basic demographic variables; and model3 adjusts for all variables in Table 1 to fully consider possible confounding factors.

**Table 1:** Baseline characteristics of study population according to SDT.

Variables	Total	7-9	<7	>9	P-value
	n=3702	n=2268	n=1226	n=208	
Age, Mean(SE)	40.40(0.24)	40.26(0.28)	41.20(0.38)	37.48(1.25)	0.01*
Race, n (%)					< 0.01*
White	1552(69.01)	1025(72.83)	444(61.39)	83(63.54)	
Black	833(11.82)	407(8.78)	382(18.37)	44(13.40)	
Mexican	679(7.89)	440(7.72)	208(8.25)	31(8.03)	
Other	638(11.28)	396(10.67)	192(11.99)	50(15.03)	
Education (%)					< 0.01*
Under high school	688(11.97)	388(10.08)	250(14.84)	50(19.97)	
High school or equivalent	777(20.24)	441(17.94)	274(23.44)	62(31.73)	
College graduate or above	2237(67.80)	1439(71.98)	702(61.72)	96(48.31)	
Marital status (%)					<0.01*
Married/Living with partner	2257(66.27)	1465(69.66)	672(59.73)	120(60.00)	
Widowed/Divorced/Separated	704(16.85)	373(14.31)	293(22.47)	38(17.27)	
Never married	741(16.88)	430(16.03)	261(17.80)	50(22.72)	
PIR, Mean(SE)	3.15(0.05)	3.32(0.06)	2.96(0.08)	2.09(0.14)	< 0.01*
BMI, Mean(SE)	28.91(0.20)	28.38(0.23)	30.13(0.27)	28.77(0.61)	<0.01*
SDT, Mean(SE)	7.18(0.03)	7.70(0.02)	5.48(0.03)	10.27(0.07)	< 0.01
Drinks (%)					<0.01*
former	533(12.92)	305(11.85)	195(14.42)	33(18.25)	
heavy	828(22.79)	464(20.81)	306(25.88)	58(30.73)	
Mild	956(27.98)	613(29.72)	301(25.74)	42(18.17)	
moderate	835(25.08)	546(26.52)	253(23.05)	36(17.94)	
never	550(11.23)	340(11.10)	171(10.90)	39(14.92)	
Smoke (%)					<0.01*
former	582(18.42)	383(19.48)	184(17.83)	15(7.94)	
Never	2279(58.54)	1466(61.44)	697(52.97)	116(53.11)	
Now	841(23.03)	419(19.08)	345(29.21)	77(38.94)	
Age of sexual debut (%)					<0.01*
<18	2108(57.04)	1216(54.48)	757(60.56)	135(70.24)	
≥18	1594(42.96)	1052(45.52)	469(39.44)	73(29.76)	
Contraceptive use (%)					0.03*
No	856(17.42)	533(17.35)	251(16.05)	72(26.36)	
Yes	2846(82.58)	1735(82.65)	975(83.95)	136(73.64)	
Female hormone use (%)					0.46
No	3250(84.31)	1998(84.33)	1057(83.40)	195(89.45)	
Yes	452(15.69)	270(15.67)	169(16.60)	13(10.55)	
OSA (%)					< 0.01*
No	2717(74.09)	1737(77.01)	826(67.99)	154(71.41)	
Yes	985(25.91)	531(22.99)	400(32.01)	54(28.59)	
HR-HPV infection (%)					< 0.01*
Negative	2932(80.61)	1838(82.71)	942(77.47)	152(71.32)	
Positive	770(19.39)	430(17.29)	284(22.53)	56(28.68)	
HPV infection (%)					< 0.01*
Negative	2066(59.07)	1350(63.01)	617(52.64)	99(44.84)	
Positive	1636(40.93)	918(36.99)	609(47.36)	109(55.16)	

PIR, poverty-to-income ratio; BMI, body mass index; SDT: sleep duration time; OSA, obstructive sleep apnea; HPV, human papillomavirus; HR-HPV, high risk human papillomavirus; *P<0.05.

Additionally, we used restricted cubic splines (RCS) to evaluate potential non-linear relationships between SDT and HPV infection, which helps to reveal more complex association patterns. To better understand the associations among different populations, we conducted stratified multivariate regression analysis and checked for heterogeneity in associations between different subgroups using interaction terms. This step helps identify unique relationships that may exist in specific populations. In all analyses, we considered a P-value of less than 0.05 as the cutoff for significance. Data analysis was performed using the R statistical software package (version 4.2.3, http://www.r-project.org). The comprehensive application of these methods allowed us to thoroughly assess the relationship between sleep factors and HPV infection, taking into account various potential influencing factors.

## Results

### Demographic characteristics of participants

Participant characteristics were described based on SDT groups (Table 1). The study included 3702 individuals (weighted number 62,094,318), of whom 2268 had an SDT of 7-9 hours (weighted number 40,807,495), 1226 had less than 7 hours (weighted number 18,199,834), and 208 had more than 9 hours of sleep (weighted number 3,086,989). Among these participants, 40.93% (weighted percentage) were infected with HPV, and 19.39% (weighted percentage) were infected with HR-HPV. There were differences in the participants’ age, race, education, marital status, PIR, BMI, alcohol consumption, smoking, age at sexual debut, hormonal drug use, OSA, HP-HPV, and HPV infection status among different SDT groups.

### Correlation between SDT, OSA and HPV/HR-HPV infection

We used weighted multivariate linear regression and/or weighted multivariate logistic regression to analyze the relationship between sleep factors (SDT, OSA) and HPV infection (Table 2). When SDT was treated as a continuous variable, we did not observe a linear relationship between the two. We then categorized SDT into discrete variables (<7 hours, 7-9 hours, >9 hours). In all three models, compared to SDT 7-9 hours, the risk of HPV infection was higher for both SDT <7 hours and >9 hours. In the fully adjusted model, SDT <7 hours had a 26.3% higher risk of HPV infection compared to 7-9 hours (OR = 1.26, 95% CI:1.029,1.549). SDT >9 hours had a 57.4% higher risk of HPV infection compared to 7-9 hours (OR = 1.574, 95% CI:1.116,2.220). A relationship between SDT and HP-HPV infection was observed in models 1 and 2, but not in model 3. OSA was not associated with HPV or HR-HPV infection (Table 3).

**Table 2:** Association between SDT and HPV/HR-HPV infection status.

Exposure	Model 1	Model 2	Model 3
	OR, 95% CI	OR, 95% CI	OR, 95% CI
**HPV infection status**			
SDT Continuous	-0.011(-0.024,0.001)	-0.009(-0.021, 0.003)	-0.004(-0.017, 0.009)
SDT Categories			
<7	1.533(1.271,1.849)	1.470(1.226,1.763)	1.263(1.029,1.549)
7-9	Reference	Reference	Reference
>9	2.096(1.600,2.745)	1.999(1.519,2.630)	1.574(1.116,2.220)
P for trend	<0.001*	<0.001*	0.003*
**HR-HPV infection status**			
SDT Continuous	-0.001(-0.012,0.011)	-0.002(-0.013, 0.010)	0.001(-0.011, 0.012)
SDT Categories			
<7	1.39(1.14,1.69)	1.39(1.13,1.72)	1.22(0.98,1.53)
7-9	Reference	Reference	Reference
>9	1.92(1.29,2.87)	1.78(1.16,2.73)	1.40(0.89,2.22)
P for trend	<0.0001	<0.001	0.04

Model 1: no covariates were adjusted.

Model 2: adjusted for age and race.

Model 3: adjusted for all variables in Table 1.

*P<0.05.

**Table 3:** Association between OSA and HPV/HR-HPV infection status.

Exposure	Model 1	Model 2	Model 3
	OR, 95% CI	OR, 95% CI	OR, 95% CI
HPV infection status			
OSA			
No	Reference	Reference	Reference
Yes	1.19(1.02,1.40)	1.24(1.05,1.46)	1.16(0.99,1.37)
HR-HPV infection status			
OSA			
No	Reference	Reference	Reference
Yes	0.98(0.77,1.24)	1.07(0.84,1.36)	1.03(0.81,1.32)

Model 1: no covariates were adjusted.

Model 2: adjusted for age and race.

Model 3: adjusted for all variables in Table 1.

*P<0.05.

### The U-shaped relationship between SDT and HPV infection

**Fig 2:**
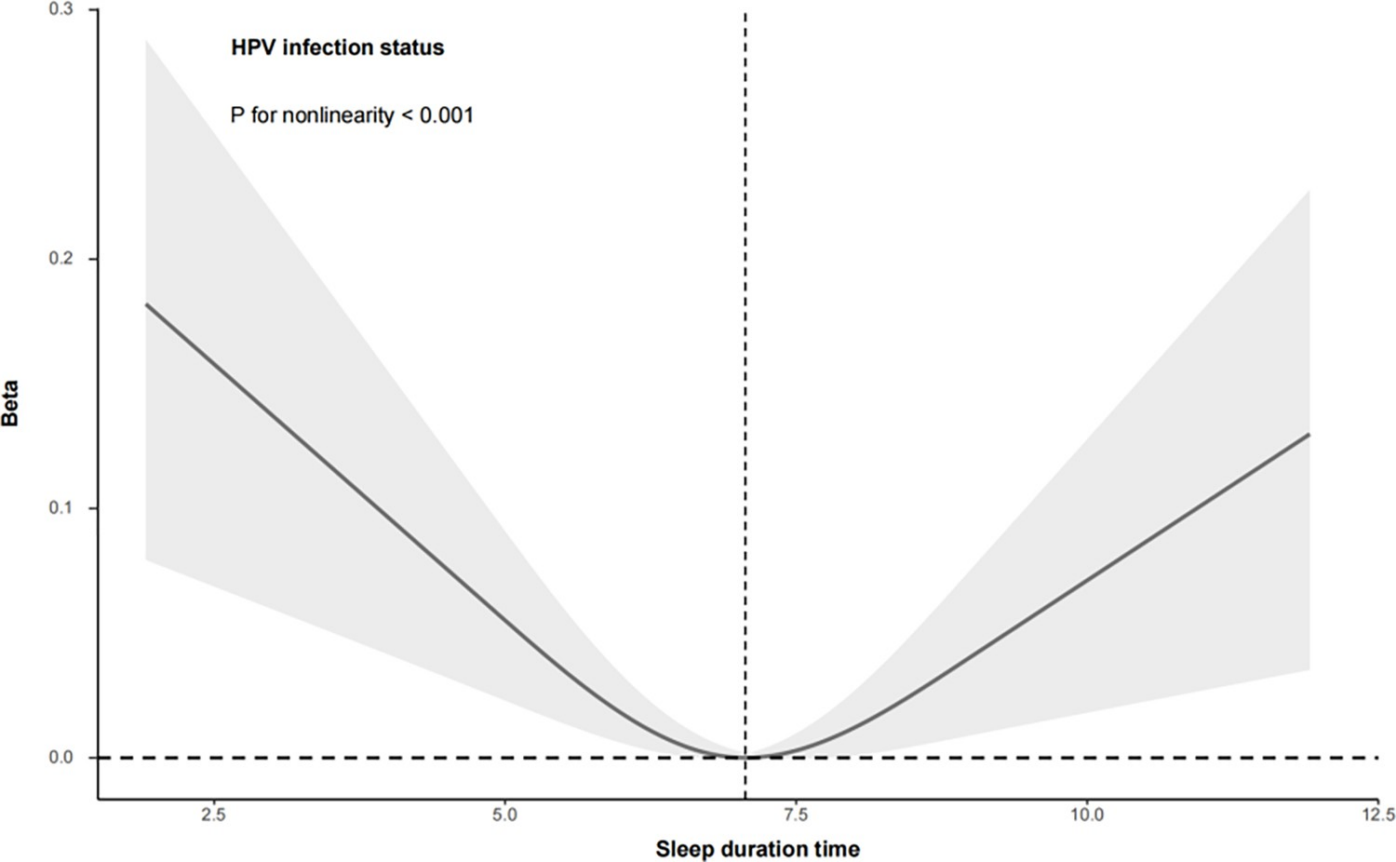
The U-shaped relationship between SDT and HPV infection.

Our study investigated the relationship between SDT and the risk of HPV infection using RCS analysis. The visual representation of this relationship is presented in Fig 2. The results demonstrate a clear U-shaped pattern in this relationship. Specifically, at shorter SDT, there is a gradual decrease in the risk of HPV infection as SDT increases. However, once SDT exceeds 7 hours, the risk of infection paradoxically increases with further increases in SDT. This suggests that there is a turning point in the trend of HPV infection risk around a specific SDT (i.e., 7 hours), resulting in a U-shaped curve. Further statistical analysis confirmed the statistical significance of this U-shaped relationship (non-linear test P value: <0.001).

### Subgroup analysis and interaction test

**Fig 3:**
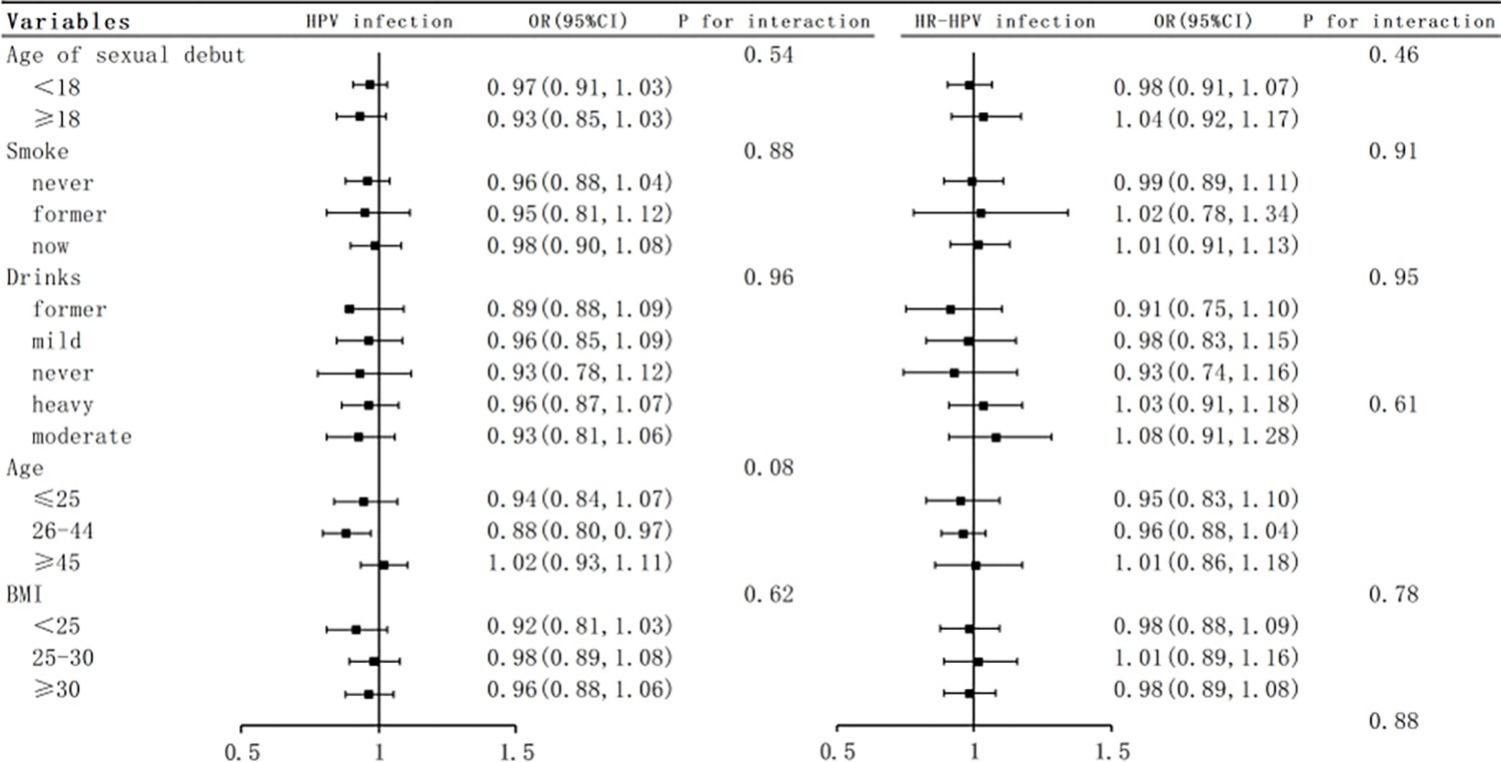
Forest plot of subgroup analysis on SDT and HPV infection.

Subsequently, we conducted subgroup analyses using explicit stratification criteria based on factors such as age at first sexual intercourse, age, smoking, alcohol consumption, and BMI. The selection of these stratification criteria was based on their potential influencing factors and relevant research. The analysis results are detailed in Fig 3. We found that the relationship between SDT and HPV infection remained statistically significant in all multivariate logistic regression models. Notably, we did not observe any significant interactions in our analysis (all interaction P-values were greater than 0.05), suggesting that the association between SDT and HPV infection may not be significantly influenced by the aforementioned variables, thereby enhancing the credibility of this finding.

## Discussion

Our cross-sectional study results revealed an association between SDT, OSA, and HPV/HR-HPV infection in the study population. Further analysis showed a U-shaped relationship between SDT and HPV infection, with the optimal sleep duration on weekdays being 7 hours. This suggests that both excessively long and short sleep durations may increase the risk of HPV infection. We speculate that this U-shaped relationship may be related to the impact of sleep on the immune system, particularly in maintaining immune balance. Our findings are consistent with other studies on the U-shaped association between SDT and health outcomes, such as previous studies that have found a similar U-shaped relationship in the risk of cardiovascular and metabolic diseases [16, 17]. These consistencies enhance the significance and reliability of our findings, while suggesting that sleep duration has a broad impact on various health outcomes.

Insufficient sleep is a recognized factor related to immune dysfunction and has been associated with increased susceptibility to various infections [18]. For instance, patients with sleep disorders have an increased risk of developing shingles, and an international collaborative study during the COVID-19 pandemic indicated that longer sleep duration is associated with a lower likelihood of COVID-19 infection [19, 20]. Specifically, the relationship between short sleep duration and increased HPV infection risk may involve the following mechanisms: Impaired T cell effector activity: Sleep deprivation is primarily characterized by a reduction in T follicular helper cells (Tfh), and the decrease in Tfh cells directly affects the maturation and function of B cells, leading to a reduction in infection-related IgG [21, 22]. This reduction could weaken the body’s immune response to HPV, increasing the risk of infection. Chronic inflammation: Sleep deprivation increases the secretion of pro-inflammatory cytokines, leading to a sustained pro-inflammatory state [23]. This continuous inflammatory state may interfere with normal immune function, making the body more susceptible to viral infections, including HPV. Cervicovaginal microbiota disruption: Sleep deprivation, by affecting the release of stress hormones such as cortisol, can cause a “stress response” and disruption of sex hormones, potentially leading to cervicovaginal microbiota disruption [24]. These disruptions may affect the reproductive tract’s immune response and clearance ability against HPV. Studies suggest that using probiotics to achieve vaginal microbiota stability may help facilitate the clearance of HPV in women with persistent infection [25]. The combined effect of these mechanisms may explain the close connection between sleep deprivation and increased risk of HPV infection.

Our study also found that compared to short sleep duration, people with long sleep duration (over 9 hours) tend to have a higher risk of HPV infection. For example, one study showed that long sleep duration is associated with an increased probability of throat and ear infections, while another meta-analysis found no significant association between sleep duration over 9 hours and upper respiratory tract infections [26, 27]. Although these studies target different types of infections, they may imply the impact of long sleep duration on the body’s immune function. Although the exact mechanism is unclear, long sleep duration may be a sign of poor health status, which might be associated with physical frailty, thereby increasing the likelihood of infection [28, 29]. Particularly, the prevalence of frailty is higher in women than in men, and frailty has been proven to increase the risk of diseases such as urinary tract infections and sepsis [30–32]. Additionally, long sleep duration may be associated with insufficient physical activity, and a lack of physical activity has been shown to significantly increase the risk of HPV infection [33]. In summary, long sleep duration may increase the risk of HPV infection through various pathways, which requires further research to clarify.

The main strength of this study lies in the utilization of NHANES’s complex design, which is based on a nationally representative population sample. We used NHANES sample weights for analysis, allowing the results to represent the entire U.S. population, thereby enhancing the study’s broad applicability and reliability. However, this study also has some limitations. Firstly, our sleep factors (SDT and OSA) data are based on participants’ self-reports, which may be subject to recall bias and subjectivity, potentially affecting the accuracy and reliability of the data. Secondly, as the HPV vaginal swab samples were only for women under 60 years of age, we could not assess the relationship between sleep factors and HPV infection risk in older women, limiting the scope of the study’s applicability. Additionally, although we adjusted for multiple covariates to control for potential confounders, there may still be other unidentified and unadjusted factors that could affect the interpretation of the final results. Therefore, the study results should be understood and applied considering these limitations.

## Conclusion

In this cross-sectional study based on NHANES data, we focused on exploring the relationship between sleep factors (SDT and OSA) and HPV/HR-HPV infection. Our main finding is that there is a U-shaped relationship between SDT and HPV infection, showing that 7 hours of sleep is associated with a lower risk of HPV infection, while both excessively long or short sleep durations are associated with a higher risk of infection. This finding highlights the complex and potentially influential relationship between sleep duration and HPV infection risk. Therefore, we suggest that future research should delve deeper into the relationship between sleep duration and HPV infection, particularly its biological mechanisms, and should consider a broader population and more precise methods of sleep assessment. In this cross-sectional study, we found a U-shaped relationship between sleep duration and HPV infection, with the optimal sleep duration being 7 hours. These findings require further prospective studies to provide more evidence.
